# Cardiovascular Magnetic Resonance Imaging: A Prospective Modality in the Diagnosis and Prognostication of Heart Failure

**DOI:** 10.7759/cureus.23840

**Published:** 2022-04-05

**Authors:** Jalal Elmadi, Lakshmi Satish Kumar, Lakshmi Sree Pugalenthi, Mahlika Ahmad, Sanjana Reddy, Zineb Barkhane

**Affiliations:** 1 Facultad de Ciencias Médicas, Universidad Nacional Autónoma de Honduras, Tegucigalpa, HND; 2 Internal Medicine, University of Perpetual Help System Dalta, Manila, PHL; 3 Research, Ago Medical and Educational Center Bicol Christian College of Medicine, Legazpi City, PHL; 4 Internal Medicine, Ziauddin University, Karachi, PAK; 5 Medicine, Bogomolets National Medical University, Kyiv, UKR; 6 Research, Universite Hassan II Faculté de Médecine et de Pharmacie de Casablanca, Casablanca, MAR

**Keywords:** management of heart failure, heart failure knowledge, cardiac radiology, cardiac magnetic resonance (cmr), heart failure prognosis

## Abstract

Heart failure (HF) is a clinical syndrome resulting from structural cardiac remodeling and altered function that impairs tissue perfusion. This article aimed to highlight the current diagnostic and prognostic value of cardiac magnetic resonance (CMR) in the management of HF and prospective future applications. Reviewed are the physics associated with CMR, its use in ischemic and non-ischemic causes of HF, and its role in quantifying left ventricular ejection fraction. It also emphasized that CMR allows for noninvasive morphologic and functional assessment, tissue characterization, blood flow, and perfusion evaluation in patients with suspected or diagnosed HF. CMR has become a crucial instrument for the diagnosis, prognosis, and therapy planning in patients with HF and cardiomyopathy due to its accuracy in quantifying cardiac volumes and ejection fraction (considered the gold standard) as well as native and post-contrast myocardial tissue characterization.

## Introduction and background

Heart failure (HF) is a syndrome that encompasses a vast constellation of characteristic subjective symptoms (lethargy and dyspnea) and objective symptoms or physical findings (lower extremity edema, pulmonary crackles, elevated jugular venous pressure, and tachycardia) usually caused by an abnormality in the heart that alters its structure or function, leading to a reduction in the cardiac output or an increase in cardiac chamber pressures [1]. Illnesses of a cardiac origin were historically associated with ineffective treatments and poorer outcomes, a trend that although attenuated still persists today [2]. Despite advances in diagnosis and treatment, HF persists as a rising cause of morbidity and mortality while placing an ever-increasing load on healthcare systems, where the global financial burden of HF in the year 2014 was estimated at $108 billion per annum [3]. Notwithstanding some dissimilarity among reported HF prevalence (geographic, gender, and age differences), statistics illustrate that clinically significant HF is increasingly common in older adults [4,5].

Many different etiologies have been associated with HF development, hypertension being chief among these. Increased systemic pressure increases the workload that the left ventricular (LV) myocardium must withstand. In response to this added stress, the left ventricle experiences structural and functional changes to comply with the increased demand [6]. Diabetes mellitus and coronary artery disease (CAD) are also significant contributors to ventricular dysfunction. Although these are the most common etiologies, HF can be caused by multiple other pathological processes such as cardiomyopathies [6].

Myocardial offenses initiate a cascade of physiological pathways that ultimately result in an adaptive response in cardiomyocytes. The principal process by which these changes occur is through a cascade of vasoactive components that ultimately lead to vasoconstriction [7]. The main symptoms (fatigue, orthopnea, and paroxysmal nocturnal dyspnea) and physical findings (S3, gallop, and peripheral edema) that occur in HF are not specific and can be seen in a wide array of pathological processes [8,9]. While HF can be diagnosed based on its clinical manifestations, it is not possible to clinically distinguish between HF with preserved and reduced LV function. Commonly used diagnostic tests include electrocardiography (ECG) to investigate cardiac conductivity, chest radiography to dismiss pulmonary disease, echocardiography (echo) to evaluate for structural heart abnormalities, blood biochemistry (proendothelin, aldosterone, C-reactive peptide, and brain natriuretic peptide), and hematology (hemoglobin level, platelet, and lymphocyte counts). Measurement of the blood concentration of natriuretic peptides secreted by the heart is also commonly used to diagnose HF [10]. The primary treatment goals in patients with HF are to improve their quality of life, functional capacity, and clinical status while preventing hospitalization. Commonly used treatment regimens that have been shown to decrease mortality and morbidity include angiotensin-converting enzyme inhibitors, beta-blockers, and aldosterone receptor antagonists. Other drugs commonly used in select cases include diuretics, angiotensin receptor antagonists, digoxin, and angiotensin receptor neprilysin inhibitors [11].

High mortality and morbidity of HF make its accurate and early diagnosis of paramount importance. Increasing the information available to clinicians regarding cardiac structure and function leads to better health outcomes in affected patients. Over the last two decades, computed tomography (CT) and magnetic resonance imaging (MRI) have emerged as valuable tests in the clinician's repertoire, capable of vastly expanding the information available to the treating physician. Cardiac magnetic resonance (CMR) allows for improved tissue characterization and analysis of cardiac motion and performance, aiding disease management. This review aims to highlight the growing importance of CMR in the evaluation and prognostication of HF.

## Review

Overview of cardiac magnetic resonance

Historical Aspects of MRI

Imaging in medicine has evolved dramatically since X-rays were discovered over 125 years ago. The arsenal of intricate and precise tests available to today's radiologists includes ultrasound, computed and positron emission tomography, and MRI, among quite a few others. In 1973, Paul Lauterbur demonstrated how to create an image using nuclear magnetic resonance (MR); he was bestowed the Nobel Prize in Physiology or Medicine for these endeavors. The first human MR images were published in 1977, and MR techniques then took nearly five hours to acquire [12]. In the late 1980s, there was only a tiny quantity of low-strength imagers; however, by 2010, tens of thousands of high-strength MR imagers were used to perform tens of millions of examinations globally. Advances in MRI can be classified as related to hardware (magnets, coils, transmitters, and receivers) and software (pulse sequences, parallel imaging, among others) [13]. CMR imaging entered the clinical arena in the early 1980s. Initial reports indicated that MRI showed a vast spectrum of normal and abnormal cardiovascular anatomy, which led to CMR's subsequent uses being studied and developed over the ensuing decades [14].

Mechanism of MRI

The magnetic characteristics of atomic nuclei are vital in MRI, as powerful magnets are utilized to produce a magnetic field that forces protons in hydrogen atoms to align with it. Protons typically oriented randomly within the water nuclei of the tissue being investigated are aligned using an external magnetic field. A radiofrequency current is emitted through the patient, stimulating protons and causing them to strain against the magnetic field's pull [15]. The initiation of an external radio frequency (RF) energy alters the alignment (or magnetization), where RF energy is emitted as the nuclei return to their resting alignment through various relaxation processes. MRI sensors are capable of detecting the energy released as the protons realign with the magnetic field. The emitted signals are measured after a specific amount of time has passed from the first RF, which are then represented as shades of gray in a grid of pixels using the Fourier transform (a mathematical transform that decomposes functions depending on space or time into functions depending on spatial or temporal frequency). The energy released and the time it takes protons to realign varies depending on the chemical nature of the molecules and the environment [15]. The resulting image varies based on the type of tissue observed; substances that contain fewer hydrogen atoms and therefore fewer protons (ligaments and bone) appear dark while those with a higher hydrogen atom concentration (fat, cerebrospinal fluid) appear bright. The image's brightness positively correlates with the speed of proton realignment. MRI's usefulness increased when the implementation of relaxation time (the time it takes protons to emit their signal) was considered. Two types of relaxation times exist among body tissues, known as T1 and T2, with values varying between tissues [15].

Initially, there were few options available concerning imaging techniques and pulse sequences. Different images can be formed by altering the sequence of RF pulses applied. The time between subsequent pulse sequences delivered to the same slice is known as repetition time (TR). The delay between the delivery of the RF pulse and the reception of the echo signal is known as the time to echo (TE) [16]. Following an intravenous bolus of gadolinium, three time phases are considered (T1, T2, and T2*) to differentiate soft tissues based on their magnetic characteristics, characterized by relaxation times [16]. The first pass can be utilized for perfusion imaging to detect ischemia, where hypovascular areas will not improve shortly after contrast is administered. Due to slower contrast kinetics and a larger volume of distribution, contrast accumulates in sites of infarction or localized fibrosis in the late phase (five minutes after the bolus). Late gadolinium enhancement (LGE) patterns vary based on the disease process and are frequently prognostic and diagnostic significant [16].

Any modern MRI scanner may be used to image the heart, provided specific cardiac sequences have been installed. Multiple standardized protocols are used based on the medical indication, with scans taking place during various breath-holding episodes usually lasting between eight and 15 seconds, whereas a standard HF protocol takes 40 minutes [17]. The initial portion of CMR typically evaluates anatomy and function utilizing double-oblique imaging oriented to the long and short axes of the heart using steady-state free precession (SSFP) ECG gated cine imaging. Most CMR studies utilize LGE to evaluate for scar formation or cardiomyopathy; however, additional techniques can be used to provide real-time imaging during the respiratory cycle, myocardial blood flow stress, and edema imaging, among other uses. Gadolinium (an extracellular contrast agent) dramatically enhances the differences between relaxation times in normal and abnormal myocardium [18].

CMR in HF

Cardiac findings derived from CMR can uncover undiagnosed heart diseases and identify structural alterations that could negatively affect prognosis in many patients. Techniques include stress cardiac MRI, volumetric assessments, tissue characterizations, and LGE. CMR can confirm left ventricular ejection fraction (LVEF) preservation and structural changes in the atrial chambers that may aid in confirming the etiology and may alter prognosis [19]. Assadi et al. evaluated the prognostic role of CMR on myocardial scarring [19]. Their meta-analysis on nine of 97 potential studies meeting inclusion criteria found an increased incidence of arrhythmias and reduced therapeutic responsiveness. The main cardiac MRI methods that demonstrated association to prognosis in HF included LGE assessment of scar (n = 3), tissue characterization with T1-mapping (n = 4), myocardial ischemia (n = 1), and right ventricular dysfunction (RVD) (n = 1). The pooled HR for all nine studies was 1.52 (95% CI: 1.05-1.99, P < 0.01) [19]. These results support the findings published by White and Patel in a 2007 review, which concluded that CMR could provide detailed evaluations of myocardial and valvular function and morphology that can lead to a more exhaustive evaluation in HF patients [20].

The role of CMR in the assessment of patients with HF is constantly evolving. The extent of pathologic involvement that can be detected via CMR provides valuable prognostic information in patients with HF due to various etiologies, such as ischemic cardiomyopathy (ICM), dilated cardiomyopathy (DCM), iron overload, and cardiac amyloidosis [21]. CMR's use in HF is supported by findings by Peterzan et al. in a 2016 review that concluded that using different mapping sequences (T1, T2, and T2*) improves the evaluation of HF and cardiomyopathies [21]. New diagnostic uses for CMR are emerging utilizing different imaging methodologies. In a review by Lota et al. in 2017, it was reported that, given its ability to detect reversible myocardial inflammation, T2 mapping has impacted the routine clinical evaluation of patients with recent-onset HF [22]. LGE is another growing MR methodology currently being utilized for HF prognostication. A 2018 meta-analysis of 34 studies constituting 4,554 patients conducted by Becker et al. reported that the prognosis for adverse cardiovascular events in DCM is substantially worsened by the presence of LGE [23]. Patients with LGE had increased cardiovascular mortality (odds ratio (OR): 3.40; 95% CI: 2.04-5.67) and rehospitalization for HF (OR: 2.66; 95% CI: 1.67-4.24) compared with those without LGE [23]. MRI's ability to combine anatomic imaging with the evaluation of cardiac function at multiple scales (molecular, macro, and microscopic) is unsurpassed. CMR can provide clinicians with a vast breadth of information as it can discover previously unknown pathologies that could otherwise be missed by other imaging modalities [23]. This claim is supported by an observational study conducted in 2018 by Kanagala et al., which reported that CMR detected previously undiagnosed pathology in 42 patients (27%) [24]. These diagnoses consisted of CAD, microvascular dysfunction (n = 11), hypertrophic cardiomyopathy (HCM) (n = 10), and constrictive pericarditis (n = 5). During follow-up, patients with a new diagnosis were at higher risk of adverse outcomes for the composite endpoint (log-rank test: p = 0.047) [24]. A new CMR diagnosis was the strongest predictor of adverse outcomes (hazard ratio: 1.92; 95% CI: 1.07-3.45). These findings meant that patients were at increased risk of death and HF hospitalization [24].

CMR's usefulness extends beyond established HF management and prognostication. Many pathologic processes that predispose to or directly cause HF can be accurately evaluated using CMR [25]. Kwong et al. performed a prospective observational study in Boston, USA, in 2004, which determined that MRI detected a high fraction of patients with the acute coronary syndrome, including patients with unstable angina undetected using other cardiac imaging modalities. The diagnostic performance of MRI was evaluated in 161 consecutive patients [25]. MRI was performed at rest within 12 hours of presentation and included perfusion, LV function, and gadolinium-enhanced myocardial infarction detection. MRI was interpreted qualitatively but also analyzed quantitatively. The sensitivity and specificity, respectively, for detecting acute coronary syndrome were 84% and 85% by MRI, 80% and 61% by an abnormal ECG, 16% and 95% for strict ECG criteria for ischemia (ST depression or T-wave inversion), 40% and 97% for peak troponin-I, and 48% and 85% for thrombolysis in myocardial infarction (TIMI) risk score of 3. The MRI was more sensitive than strict ECG criteria for ischemia (P = 0.001), peak troponin-I (P = 0.001), and the TIMI risk score (P = 0.004), and MRI was more specific than an abnormal ECG (P = 0.001) [25]. This claim is supported by a prospective observational study conducted in Florence, Italy, in 2006 by Casolo et al., which reported that the majority of CAD patients (98%) showed LV contrast hyperenhancement with respect to non-CAD HF subjects (16%). Among HF patients, LGE detection by CMR had a sensitivity of 98%, a specificity of 84%, and an accuracy of 93% in detecting CAD etiology [26].

Hypertension is a significant cause of HF and can be present in up to 90% of patients; however, no noninvasive imaging technique has shown the same ability to identify structural differences between patients with hypertensive heart disease and HF with preserved ejection fraction (HFpEF) [27]. A 2018 prospective cross-sectional study published in the United Kingdom by Mordi et al. studied 112 patients who underwent cardiopulmonary exercise and biomarker testing, an imaging protocol including echo with speckle-tracking analysis, and CMR including T1 mapping pre- and post-contrast [27]. Global longitudinal strain (GLS) measured through echo and extracellular volume (ECV) measured by CMR were the variables independently stratified among the three groups of patients. ECV was the best technique to differentiate between hypertensive heart disease and HF (ECV area under the curve (AUC): 0.88; GLS AUC: 0.78). Using ECV, a cutoff of 31.2% gave 100% sensitivity and 75% specificity (Table 1) [27].

**Table 1:** Summary of cited studies regarding cardiac MRI's utility in the evaluation of heart failure. MRI: magnetic resonance imaging; HF: heart failure; CMR: cardiac magnetic resonance; LGE: late gadolinium enhancement; DCM: dilated cardiomyopathy; CAD: coronary artery disease; ECV: extracellular volume.

References	Design	Year of publication	Conclusion
Assadi et al. [19]	Systematic review and meta-analysis	2021	Cardiac MRI has value in the prognostication of patients with HF. Patients with a detectable scar, myocardial fibrosis, or ischemia appear to have a worse prognosis.
White and Patel [20]	Review	2007	Careful application of CMR provides an opportunity to improve diagnostic efficiency and care in HF patients.
Peterzan et al. [21]	Review	2016	CMR has an evolving role in assessing patients with HF, particularly the confirmation of underlying etiology.
Lota et al. [22]	Review	2017	CMR T2 mapping is likely to impact routine clinical evaluation of patients with heart failure, given the ability to detect reversible myocardial inflammation.
Becker et al. [23]	Meta-analysis	2018	The presence of LGE on CMR substantially worsens the prognosis for adverse cardiovascular events in DCM patients.
Kanagala et al. [24]	Observational study	2018	CMR identifies previously undetected alterations in a significant amount of patients with HF.
Kwong and Arai [25]	Prospective observational study	2004	MRI detected a high fraction of patients with acute coronary syndrome, including patients with enzyme-negative unstable angina.
Casolo et al. [26]	Prospective observational study	2006	CMR is among the most important diagnostic tools in the workup of patients with HF, and LGE can accurately differentiate CAD from non-CAD etiology of HF.
Mordi et al. [27]	Prospective cross-sectional study	2018	ECV is the best diagnostic marker of HF and can be accurately quantified by CMR.

MRI and other imaging modalities in the evaluation of LVEF

The diagnosis of HFpEF requires the following conditions to be satisfied: evidence of diastolic LV dysfunction, signs or symptoms of HF, and normal or mildly abnormal systolic LV function. The evaluation of LVEF is of utmost importance in guiding patient management. The extent of variation in the quantification of LVEF by different imaging procedures is currently a topic of great interest [28]. A 2016 review by Peterzan et al. concluded that while two-dimensional (2D) echocardiography has a superior temporal resolution for assessment of LV filling, CMR may contribute to statistically significant superior assessment of LVEF, LV mass, and left atrium (LA) volumes [21]. This conclusion is supported by a Norwegian randomized controlled trial in 2010 by Mistry et al., where standard echo, contrast echo, single-photon emission computed tomography (SPECT), and MRI were performed on the same day, three months after ST-elevation myocardial infarction (STEMI) in 150 patients, which reported that all four imaging modalities measured EF similarly after STEMI [28]. Bland-Altman analysis of EF measured by all four imaging modalities generally showed statistically significant low mean differences but wide limits of agreement. The mean end-diastolic volume (EDV) difference, however, was consistently higher when MRI was compared with standard echo (54.9 mL), contrast echo (41.7 mL), and SPECT (54.6 mL). The mean EDV differences between contrast echo vs. standard echo, SPECT vs. standard echo, and contrast echo vs. SPECT were small [28]. The optimal cardiovascular imaging modality varies based on the information required, exemplified by a 2013 review by Marwick et al., which concluded that CMR is the reference method for LV and right ventricular (RV) anatomy and function, while echo is superior for valvular and hemodynamic evaluation, where slight differences in critical findings can drastically alter management [29]. The mean difference in LVEF measurement between echo and CMR has been estimated to be 4%, with LVEF as measured by CMR being more predictive of mortality due to echo's slight overestimation of LVEF leading to placement in better functional categories [29]. In a 2014 observational study by Gouda et al., it was concluded that CMR is the favored technique for volume and ejection fraction (EF) estimation when resources permit, as echo yields higher LVEF values that lead to assorting patients in better functional categories [30]. The study included 152 patients (106 male, mean age: 65.5 ± 9.9 years) referred for device therapy (pacemakers, cardiac resynchronization devices, and implantable cardioverter-defibrillators). They underwent both CMR and echocardiographic LVEF assessment during the evaluation of eligibility, where CMR volumes were computed from a stack of short-axis images, and echocardiographic volumes were computed using Simpson's biplane method [30]. The study population demonstrated an underestimation of EDV and end-systolic volume (ESV) by echocardiography of 71 ± 53 ml (mean ± SD) and 70 ± 49 ml, respectively. This resulted in an overestimation of LVEF of 6.6 ± 8.3% by echocardiography compared with CMR (echocardiographic LVEF: 31.5 ± 8.7% and CMR LVEF 24.9 ± 9.6%) [30]. Similar findings were reported in a 2022 review by Lahoti et al., which estimated the mean difference in LVEF measurement between echo and CMR to be 4%, with LVEF as measured by CMR being more predictive of mortality [31].

CMR in cardiomyopathies

HF is caused by a loss of functional myocardial cells after injury to the heart from various causes. The most common etiologies are broadly classified as ischemic or non-ischemic cardiomyopathies [31]. According to epidemiological studies and large-scale treatment trials, patients with ischemic HF have a worse prognosis than those with non-ischemic etiologies. ICM refers to the heart's reduced ability to pump blood correctly due to ischemia-induced myocardial damage, with CAD being the most common contributor. Myocardial infarction is characterized by a non-contractile myocardium secondary to an ischemic insult and is the most frequent cause of death in industrialized countries [32]. After approximately 40 minutes of ischemia, the adenosine triphosphate (ATP) storage in cardiomyocytes is fully depleted. ATP deficiency halts most cellular metabolic processes resulting in the accumulation of toxic metabolites and ultimately leading to cell death. The maximum extent of the infarcted tissue is reached approximately six hours after the onset of ischemia [32].

Initial MR studies reported that myocardial contrast enhancement in infarcted regions was clinically relevant. Areas of myocardial necrosis appear as hyper-enhanced myocardial regions, while it was possible to visualize aspects such as the no-reflow zone, the border zone of ischemic injury, and the centrifugal gradient of necrosis [32,33]. Today, CMR is recognized as a method of high spatial resolution for interpreting myocardial injuries due to its lack of ionizing radiation, non-invasiveness, and the excellent safety profile of currently used contrast agents [33]. Several MRI techniques have been developed to characterize the heart adequately. Unenhanced CMR can quantify wall motion and thickening, ventricular EF, and distinguish morphologic changes. Perfusion MRI of the myocardium can demonstrate enhancement patterns that signify decreased myocardial tissue perfusion [34]. Cine imaging is the basic imaging technique for assessing ventricular function, while MRI angiography is noninvasive and can provide valuable coronary artery imaging. LGE-CMR can identify individuals suspected of having chronic or acute ischemic heart disease and the extent and location of myocardial necrosis [34]. CMR has high diagnostic accuracy for detecting CAD, the principal cause of ischemic heart disease. This claim is supported by a 2014 comparative study in the USA by Mordini et al., in which dual bolus dipyridamole stress perfusion CMR exams were performed in 67 patients with clinical indications for assessing myocardial ischemia [35]. Stress perfusion images alone were analyzed with the fully quantitative perfusion (QP) method, and three semi-quantitative methods (contrast enhancement ratio, upslope index, and integral) with a 70% or greater stenosis by quantitative coronary angiography were considered abnormal. The optimum diagnostic threshold yielded a sensitivity of 87% and specificity of 93% [35]. QP AUC was 92%, superior to semi-quantitative methods, upslope index was 82%, contrast enhancement ratio was 78%, and upslope integral was 75% (p = 0.011, p = 0.019, p = 0.004 vs. QP, respectively) [35].

Non-ischemic cardiomyopathy (NICM) comprises a wide range of primary and secondary (due to a systemic disease) heart pathologies and commonly causes HF, arrhythmias, and sudden cardiac death (SCD) [36]. NICM includes acquired forms (myocarditis, stress-induced, and peripartum cardiomyopathy), genetic forms (HCM, LV noncompaction, and others), as well as mixed forms (dilated and restrictive cardiomyopathies) [36]. CMR represents a noninvasive measure to determine chamber size and structure, tissue composition and metabolism, and ventricular function and perfusion in these patients. This information is vital in identifying the etiology and also aids in establishing therapy and prognosis [37]. Several CMR sequences are commonly used in the evaluation of NICM. The most common sequence, SSFP, aids in evaluating ventricular morphology and function [38]. Velocity-encoded phase-contrast MR can quantify flow and velocity in cardiac structures, and cardiac edema can be detected using T2-weighted images. Myocardial iron can be quantified using multi-echo graded images, and different LGE patterns are used to show myocardial fibrosis and scar tissue [38]. CMR's use in evaluating cardiomyopathies is steadily increasing due to its exceptional accuracy [39]. A 2019 systematic review by Mayala et al. conducted in China with data acquired from January 2013 to April 2017 that included 12 studies reported that CMR's average sensitivity and specificity in the diagnosis of cardiomyopathy was 86.75% (95% CI), and the positive predictive and negative predictive values were 80.17% and 86.75%, respectively [39].

Practicability of CMR

Validity and Feasibility

Despite the safety of most modern radiological procedures, the absence of radiation and the existence of relatively safe contrast media increase the likelihood of patients undertaking the test. This is in stark contrast to the invasiveness and radiation exposure commonly experienced in other imaging modalities such as coronary angiograms, positron emission tomography, and best radiography [29]. While cardiac CT can be performed utilizing low-dose radiation, evaluating vital parameters such as LV size and function requires higher radiation doses. Due to this, the complete evaluation of cardiac disease in large populations is best served by utilizing CMR and echo. The validity of tests is increased when testing is possible in the largest number of patients; due to this, CMR's validity is steadily increasing [29,40].

Accuracy

Although not applicable to all metrics, CMR has an advantage over other tests in terms of accuracy. There are important differences in the accuracy of CMR as opposed to 2D echo measurements of LV mass and volume [40]. A 2001 double-blinded, placebo-controlled clinical trial in Germany conducted by Strohm et al. that studied 50 patients with markedly reduced LVEF reported an interstudy difference of EF of 24 ± 18%, compared with only 17 ± 19% with CMR [40]. LV dimensions and wall thickness did not differ significantly between 2D echo and MRI. In contrast, there were significant differences between the 2D echo calculations and the MRI measurements for the three-dimensional (3D) parameters: LV-EF was significantly higher in 2D echo than in MRI [40]. This claim is supported by a 2011 comparative study by Crean et al. in Canada, where Bland-Altman analysis of 25 patients demonstrated a significant and systematic under-estimation for RV EDV and RV ESV of volume by 3D echo compared to CMR [41]. This led to a mean underestimation of RV EDV by −34% (95% CI: −91% to +23%). There was a tendency to overestimate RV EF by 3D echo with a bias of approximately 13% (95% CI: −52% to +27%). Due to the low variance across multiple CMR measurements, this technique has been chosen for patient evaluation in some clinical studies over alternative LV evaluation strategies such as echo [41].

Limitations of CMR

Although CMR has proven to be a valuable tool in diagnosis while offering several advantages over other modalities, certain limitations and challenges are still present. More information can sometimes lead to diagnostic confusion, as incidental findings can trigger anxiety and further testing [29]. CMR's widespread use is limited by several factors: lack of availability, long acquisition time, parietal volume effects, cost, and contraindication in patients with metallic implants and other non-MR-compatible devices (cerebrovascular clips or metallic objects in the eye). However, technological advancements in pacemaker compatibility with MRI are expected [29,41,42]. CMR requires a cardiac dedicated scanner and is more expensive than echocardiography. Evaluation of patients with tachyarrhythmias or breathing artifacts is limited due to unreliable measurements; however, free-breathing techniques involving T2/T2*-weighted images can potentially save time. Extended scan times also pose a problem in the context of acute myocardial infarction (AMI), especially for those with poor LV function or large infarct size. Due to this, a shortening of scan times is necessary to make CMR practicable in the evaluation of AMI [42]. The widespread use of CMR is challenging in some populations, such as those with cognitive impairment and limited mobility; those in socioeconomically depressed or rural areas can also have significant difficulties obtaining evaluation. Although portable MR scanners exist, most environments outside healthcare settings are unsuitable for CMR or lack the infrastructure to support it adequately. An accurate understanding of the techniques employed is vital for MRI's proper utilization, and its underlying complexity has hindered many clinicians from utilizing it fully [43]. Claustrophobia is associated with an increased likelihood of study failure, although its incidence can be decreased by utilizing recently developed MR scanners and benzodiazepines [44]. Gadolinium-based contrast is contraindicated in patients with renal dysfunction with a glomerular filtration rate (GFR) of less than 30 ml/min/m2 due to the possibility of developing nephrogenic systemic fibrosis, a rare complication of LGE and dialysis [43].

Applications of CMR

The Society for Cardiovascular Magnetic Resonance (SCMR) lists the situation in which CMR use obtains a Class I classification, including general evaluation of RV and LV volumes, mass, and function, and measurement of the pulmonary-to-systemic flow ratio [45]. CMR has a Class I recommendation in the evaluation of several shunt lesions (sinus venous defects, anomalous pulmonary venous connection, and systemic-to-pulmonary artery collaterals), arterial lesions (vascular rings), conotruncal lesions (truncus arteriosus, RV double outlet, and transposition of the great arteries) as well as complex diseases such as heterotaxy syndrome and single ventricle heart disease. The SCMR gives many critical pathological processes a Class II classification; these include the initial evaluation and follow-up of congenital heart disease and the evaluation of valve lesions (tricuspid, pulmonary, and aortic valve disease). As CMR protocols improve, more medical indications should be elevated to Class 1 recommendations (Table 2) [45].

**Table 2:** The practicality of cardiac magnetic resonance (CMR) in specific diseases is summarized in the following classification.

Class	Definition
Class I	Provides clinically relevant information and is usually appropriate; may be used as a first-line imaging technique; usually supported by substantial literature or randomized controlled trial(s).
Class II	Provides clinically relevant information and is frequently useful; other techniques may provide similar information; supported by limited literature.
Class III	Provides clinically relevant information but is infrequently used because the information from other imaging techniques is usually adequate.
Class IV	Potentially useful, but still investigational.

Future implications of cardiac MRI

CMR has established itself as an essential modality in evaluating HF and cardiomyopathies. Compared with other imaging techniques (nuclear scintigraphy, coronary angiography, and echocardiography), the clinical use of cardiac MRI in heart disease is rising [30]. CMR is recognized as a method of high spatial resolution for interpreting myocardial injuries due to its noninvasiveness and the excellent safety profile of currently used contrast agents [30]. CMR's versatility may lead to its integration into different interventional procedures due to the highly accurate structural and functional information it provides [30]. CMR's many advantages include high-quality spatial and temporal images that are non-operator dependent regardless of body size and freedom from ionizing radiation, making it the ideal modality for evaluating young patients and those who require more frequent imaging follow-ups [28]. The high resolution of the images provided allows for the acquisition of superior functional parameters. CMR is evolving from simply an initial diagnostic tool to one whose findings can also have a significant clinical impact. Therapy response, risk stratification, and prognosis determination are just some of its current uses, with more potentially on the way [34]. MRI incorporates a multidisciplinary team whose combined efforts continue to extend this technique's usefulness and effectiveness. The last decade has seen enormous technological advances in CMR hardware and software. It is expected that future developments with tracers and targeted contrast media will enable the characterization of even more cellular and molecular derangements that will likely prove helpful in clinical practice [43]. The clinical potential of newer functional MR techniques (MR elastography, molecular imaging, among others) is just beginning to be exploited. The growing regard for CMR as an ideal imaging modality in several clinical settings compounded with ever-increasing accessibility indicates its use in medicine will only increase in the ensuing decades [35,41,43].

Limitations

Errors in the acquisition were minimized by following standard guidelines, although variability can occur in the acquisition and data analysis. However, this study has two limitations. This study does not address the fact that the availability of MRI, due to its high cost and size, is more common in larger urban centers and may not be available for diagnostic use in smaller hospitals, limiting its use. This study does not delve deeply into the multitude of different pathological processes where CMR may be utilized but instead provides a general overview of its possible use in multiple etiologies of HF.

## Conclusions

As evidenced by the studies reviewed in this article, CMR has proven to be a reliable and essential tool in the complete assessment of HF. Cardiac remodeling universally occurs in all HF etiologies; CMR can detect cardiac alterations such as fibrosis and hypertrophy early and accurately in many instances. CMR-based detection of HF and its pathogenesis can aid in early medical therapy initiation in symptomatic and asymptomatic patients at risk for HF. Image-based, multidimensional, patient-specific CMR models created for HF utilization that combine characterizations of myocardial deformation, tissue microstructure, and intracardiac flow data are forthcoming. CMR has the potential to be a "one-stop-shop" for HF evaluation as it can be used in the thorough assessment of all new and established cases of HF. The clinical implication of this article is to establish the growing importance of CMR in the adequate characterization and subsequent management of HF. It is critical that general practitioners and specialists alike are aware of these techniques so they can consider these advancements for the benefit of their patients. We believe this article can benefit clinicians by providing a concise description of a growing modality in evaluating a common yet grievous pathology. Despite its evident advantages, widespread CMR use is limited in large part due to a lack of accessibility. Unless comparative-effectiveness studies with clinical outcome data and market metrics are widely available, the demand and access to CMR will remain limited to patients in large medical centers. We feel that HF patients and the medical field, in general, will benefit significantly from continued research investigations into the many current and future applications of CMR to organize a more efficient approach to cardiac pathology and HF in particular.
